# Response to Comment “Sex-specific exposures and sex-combined outcomes in two-sample Mendelian randomization may mislead the causal inference” on “Age at menarche, age at natural menopause, and risk of rheumatoid arthritis—a Mendelian randomization study”

**DOI:** 10.1186/s13075-022-02923-6

**Published:** 2022-10-26

**Authors:** Jingjing Zhu, Zheng Niu, Lars Alfredsson, Lars Klareskog, Leonid Padyukov, Xia Jiang

**Affiliations:** 1 The First Affiliated Hospital of Zhejiang Chinese Medical University, Hangzhou, China; 2 Department of Gynecology, Affiliated Hangzhou First People’s Hospital, Zhejiang University School of Medicine, Hangzhou, China; 3 Department of Clinical Neuroscience, Center for Molecular Medicine, Karolinska Institute, Tomtebodavägen 5, 17 177 Stockholm, Sweden; 4 Center for Molecular Medicine, Karolinska Institute, Stockholm, Sweden; 5 Program in Genetic Epidemiology and Statistical Genetics, Department of Epidemiology, Harvard T.H. Chan School of Public Health, Boston, USA; 6 West China School of Public Health and West China Fourth Hospital, Sichuan University, Chengdu, China

## To the editor

We Would Like to Express Our Sincere Gratitude to Dr Wang Et Al. for Their Interest On Our Work As Well As Their Critical Appraisal to Our Publication Entitled “Age at Menarche, Age at Natural Menopause, and risk of rheumatoid arthritis — a Mendelian randomization study”.

In their comment, the authors pointed out a core issue, the sex-heterogeneity, which is not uncommon in Mendelian randomization (MR) design and might also bias the causal estimates of MR analysis. When the exposure of interest can only occur in one sex (e.g., prostate cancer, age at menarche, or age at nature menopause), ideally one would want the associations between SNPs and outcome estimate to be sex specific. First, we appreciate this feedback and acknowledge the potential bias possibly yielded by sex-specific and sex-combined data. Meanwhile, we would also like to point out that the magnitude of such bias might be small. Indeed, the genome-wide genetic data (GWAS) of rheumatoid arthritis (RA) was generated based on sex-combined samples involving both men and women, yet women predominated the sample with a proportion of > 70% [1]. We would therefore consider the RA GWAS as a female-dominated GWAS.

Moreover, even when the similarity assumption of age and sex distribution between gene-exposure and gene-outcome associations is violated, MR approach could still provide evidence on whether a causal association exists despite not necessarily on the precise magnitude of the causal effect [2]. Future work on such topics may be focused on different sex categories.

The author also pointed out we might have misused sex-combined summary-level GWAS data of age at first birth (AFB) in our study. Here, first we would like to apologize for the confusion we made due to misuse. We have double-checked the data and replaced sex-combined genetic instruments with female-specific genetic instruments from which we generated similar non-significant results. The result of inverse-variance weighted approach (OR = 0.74, 95%CI = 0.55–1.01) even contradicted that of MR-Egger (OR = 10.83, 95%CI = 0.01–1.48 × 10^4^). The results of multi-variable analysis conditional on body mass index and year of education, as well as sensitivity analysis also remained largely non-significant. The number of genetic instruments in sex-specific AFB subset decreased to 6 compared to 10 in our previous sex-combined subset, another reason for the non-significance (an increased sex-homogeneity but a decreased power). Future large-scale well-designed MR should be implemented to understand the effect of hormonal reproductive factors and risk of RA.
